# Superomedial-Posterior Pedicle-Based Reduction Mammaplasty: Evaluation of Effectiveness and BREAST-Q Outcomes of a Rapid and Safer Technique

**DOI:** 10.1007/s00266-023-03676-6

**Published:** 2023-10-02

**Authors:** Simone La Padula, Thibaud Mernier, Quentin Larcher, Chiara Pizza, Francesco D’Andrea, Rosita Pensato, Jean Paul Meningaud, Barbara Hersant

**Affiliations:** 1 Department of Plastic and Reconstructive Surgery, Università degli studi di Napoli Federico II, Via Pansini 5, 80131 Napoli, Italy; 2 Department of Plastic, Reconstructive and Maxillo facial Surgery, Henri Mondor Hospital, University Paris XII, 51 Avenue du Maréchal de Lattre de Tassigny, 94000 Créteil, France; 3 50 rue Saint Sébastien, 75011 Paris, France

**Keywords:** Breast hypertrophy, Breast reduction mammoplasty, Breast Q, Nipple–areola complex (NAC) sensitivity, Suction drains, Operative time in breast reduction mammaplasty

## Abstract

**Introduction:**

Breast hypertrophy, a common pathological condition, often requires surgical intervention to alleviate musculoskeletal pain and improve patients’ quality of life. Various techniques have been developed for breast reduction, each with its own advantages and complications. The primary aim of this study is to evaluate the efficacy, safety, and patient-reported outcomes of the authors technique: the Superomedial-Posterior Pedicle-Based Reduction Mammaplasty.

**Material and Methods:**

A prospective study was conducted on 912 patients who underwent breast reduction surgery between November 2012 and July 2020. The surgical technique involved preserving all glandular tissue from the areola to the pectoralis major muscle using the superomedial-posterior pedicle. The patients’ demographic data, operative details, complications, breast-related quality of life (measured using the Breast-Q questionnaire), and nipple–areola complex sensitivity were analyzed.

**Results:**

The average operative time was 62.12 ± 10.3 minutes. Complications included minor wound dehiscence (4.05%) and hematoma (1.2%), with no cases of nipple–areola complex necrosis. Nipple–areola sensitivity was fully restored in all patients at the 2-year follow-up. Patient satisfaction with the procedure was high with a statistically significant difference observed between pre- and postoperative scores (*p* < 0.001) of the Breast-Q questionnaire.

**Conclusion:**

Authors technique offers reliable vascularization and innervation of the nipple–areola complex and achieves satisfactory aesthetic outcomes. It is associated with shorter operative times compared to other techniques reported in the literature. The Superomedial-Posterior Pedicle-Based Reduction Mammaplasty represents a safe and effective method for breast reduction surgery, providing significant benefits to patients with breast hypertrophy.

**Level of Evidence I:**

This journal requires that authors assign a level of evidence to each article. For a full description of these evidence-based medicine ratings, please refer to the Table of Contents or the online Instructions to Authors www.springer.com/00266.

**Supplementary Information:**

The online version contains supplementary material available at 10.1007/s00266-023-03676-6.

## Introduction

Breast hypertrophy is a prevalent pathological condition that can lead to disability due to musculoskeletal pain resulting from the excess weight of the breasts. Managing this condition through plastic surgery interventions is a common practice that offers both psychological and physical relief to patients, significantly improving their quality of life [1–3]. Several techniques have been developed to address breast hypertrophy [4, 5]. These techniques rely on distinct pedicles, including the superior pedicle [6], superomedial pedicle [7], and other variations in pedicle choice. They have undergone extensive research, revealing varying rates of complications. Complications associated with the superomedial pedicle technique can range from 1.6 to 43% [7]. These varying rates of complications can be attributed to various factors, including both intrinsic factors such as smoking, diabetes, or hypertension, and extrinsic factors such as prolonged operating times exceeding 199 minutes [8]. Preserving all the glandular tissue from the areola to the pectoralis major muscle may help reduce complication rates by ensuring better vascularization and innervation of the nipple–areolar complex (NAC).

The primary aim of this study is to evaluate the effectiveness, safety, and BREAST-Q outcomes of the authors’ technique: the Superomedial-Posterior Pedicle-Based Reduction Mammaplasty.

The secondary objective is to evaluate the complication rate based on the utilization or absence of suction drains and the quantity of removed tissue.

## Material and Methods

We conducted a prospective study on patients who sought breast reduction surgery for large and ptotic breasts at our university hospital between November 2012 and July 2020. All patients underwent two preoperative consultations to discuss the resulting scars, potential complications, and the details of the procedure.

The study’s inclusion criteria were as follows: participants had to be aged 18 or older, in the case of active smokers, only those who agreed to cease smoking for a minimum of six weeks before the surgery and one month after were included.

To ensure compliance, all patients with a history of smoking were tested 15 days before and the day before the surgery using Screen Pharma Check, a rapid test for the qualitative detection of cotinine (a nicotine metabolite) in human urine. If they tested positive, they were excluded from the study. Additionally, patients with a previous smoking history were monitored after surgery to assess their abstinence from smoking. Qualitative detection of cotinine in human urine was repeated in these patients 15 days and 30 days after the procedure. If they tested positive, they were excluded from the analysis.

The exclusion criteria encompassed individuals with uncontrolled diabetes (determined through glycated hemoglobin examination), those unable to quit smoking, obese individuals (with a BMI equal to or exceeding 30), and patients with cognitive deficits impeding comprehension and consent.

All eligible patients were informed of the risks and benefits of the proposed procedure, received detailed information about the study, and provided written informed consent to participate.

Furthermore, all patients underwent preoperative mammography and ultrasound scans to identify any possible lesions prior to surgery. The procedures performed in the study were in concordance with the ethical standards of the institutional and/or national research committee and the 1964 Declaration of Helsinki and its subsequent amendments or comparable ethical standards.

*The Superomedial-Posterior Pedicle-Based Reduction Mammaplasty* (Video 1)*.*

Preoperative drawings:

1. The drawings were made with the patient in an upright position in their room.
2. The midline and existing inframammary creases were marked.
3. The axis of each breast was traced by connecting a point located 5 cm from the midline on the sternal notch to the midpoint of the breast. This axis was projected onto the inframammary crease.
4. The position of the future upper point of the areola was projected onto the breast axis in alignment with the existing inframammary crease. This point typically aligned with the midpoint of the humerus. The keyhole round pattern was then drawn while maintaining the natural harmony of the patient’s breast. This pattern determined the future size of the areola.
5. The vertical line was drawn by displacing the breast on both sides of its axis. A line was drawn from the inferior part of the keyhole round pattern to the projection of the breast axis on the inframammary crease. This maneuver was performed internally and externally.
6. The desired length of the vertical lines was measured on these axes. In our experience, this length typically varied between 60  and 65 mm.
7. The area of resection was then outlined. The breast was displaced until the desired shape was achieved. Once achieved, a line was drawn connecting the distal end of the vertical line to the inframammary crease both medially and laterally. To prevent the formation of visible scars, we always aimed to keep the horizontal scar at least 3 cm away from the midline.

Operative technique:

All procedures were performed under general anesthesia. We did not use any type of infiltration for the breasts. The patient was positioned in a semi-sitting position. Intraoperative antibiotic prophylaxis with 2 g of Cefazolin was administered.

**Fig. 1:**
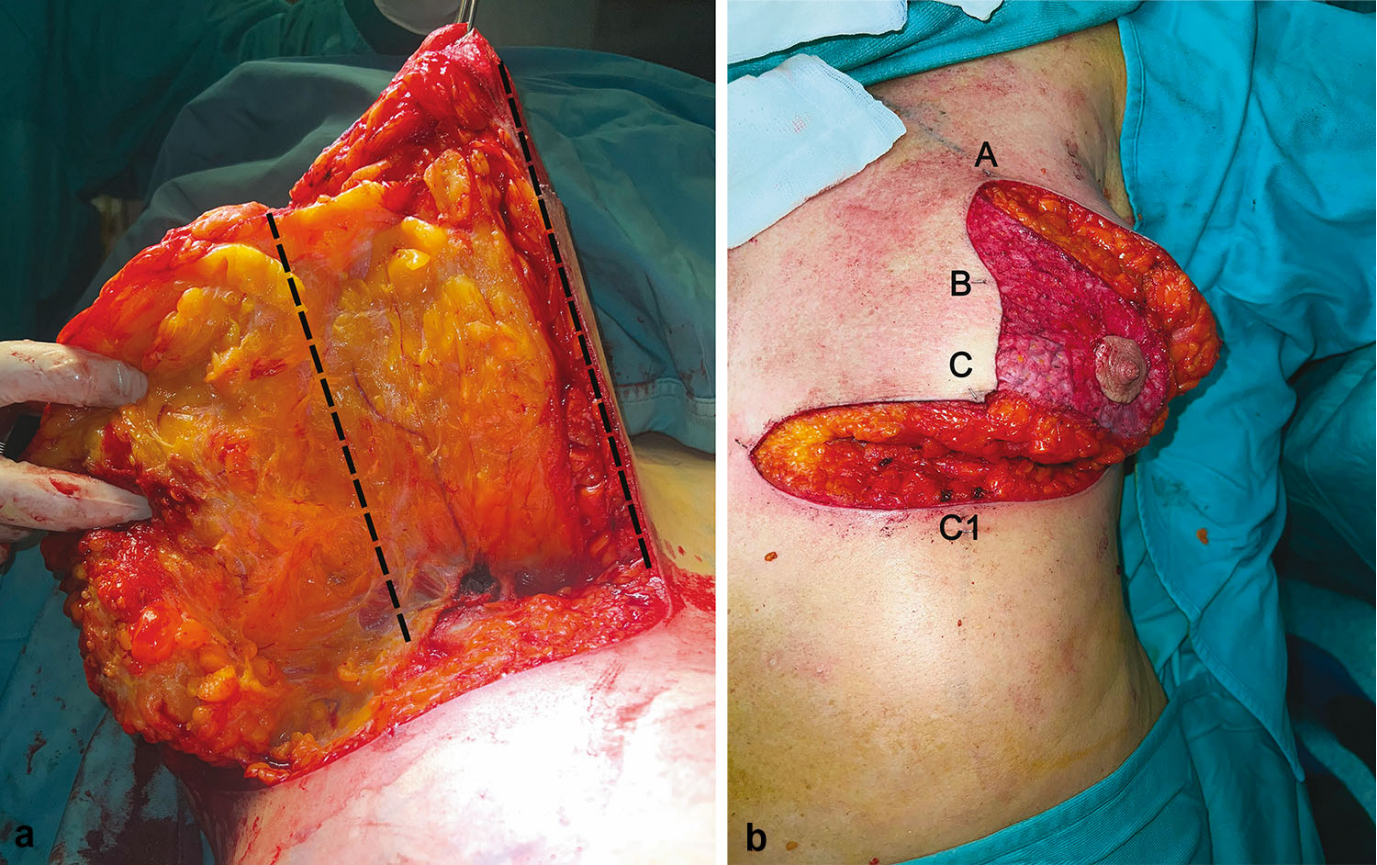
The Superomedial-Posterior Pedicle (**a**-**b**). The superomedial-posterior pedicle technique preserves all breast tissue from the nipple–areolar complex to the pectoralis muscle, ensuring a triple vascular supply. This is achieved through two vascular axes associated with the superomedial pedicle, as well as septal vascularization. The area between the two dashed lines illustrates the superomedial-posterior pedicle. In this area, the course of multiple perforating vessels that vascularize the nipple–areolar complex (NAC) can be observed (**a**). Appearance of the pedicle after the desired amount of tissue removal has been performed (**b**). By utilizing this pedicle, even in cases where there is a considerable distance between the jugulum and the nipple, and a substantial elevation of the areola is required, the vascularization and innervation of the nipple–areolar complex is preserved

1. The procedure was performed by a senior surgeon and a resident who operated on the other breast simultaneously. Incisions were made using a 15-blade scalpel, extending through the full thickness of the dermis, to ensure that the drawings lines would remain visible during the initial stages without fading.
2. The mammostat (mammostat breast elevator) was placed, and the flap carrying the nipple was de-epithelialized [11, 12].
3. Resection started externally from the pedicle, removing an appropriate amount of tissue for the desired resection.
4. The mammostat was removed, and the incisions were completed down to the subcutaneous tissue.
5. The skin-glandular-adipose tissue resection “en bloc” of the lower area relative to the pedicle (which was fully preserved from the dermis to the pectoral plane) was performed using high-intensity electric scalpel from internal to external. All the glandular tissue extending perpendicularly from the areola to the pectoralis major muscle was preserved (Figure 1). To perform this step quickly, the assistant grasped the dermis of the pedicle with a Kocher forceps and pulled the entire pedicle upward. This allowed for “en bloc” removal of the lower portion of the mammary gland to be efficiently and effectively excised while preserving the pedicle. The resections were performed by the same surgeon on each side to ensure symmetry.
6. Hemostasis was achieved.
7. A suture thread was placed on the external side of the areola, which rotated it externally and upward by 90 degrees to be sutured to the cranial portion of the breast axis (A-point).
8. A temporary suture was made using non-absorbable sutures and staples to facilitate symmetry control.
9. In the suction drains group, drains were used and removed when the volume over 24 hours was less than 30mL.
10. Once symmetry was achieved, skin edges were reapproximated via subcutaneous everting sutures and an intradermal running suture using only Monocryl 3-0 (Ethicon, Johnson and Johnson).
11. A compressive dressing (Velpeau crêpe bandage) was applied and removed on postoperative day 1. Subsequently, a supportive compression bra was to be worn for 6 weeks. All procedures were performed by the first (SLP) and last authors (BH).

Outcome assessment was conducted using the Breast-Q questionnaire [9] and the evaluation of nipple–areola complex (NAC) cutaneous sensitivity. In 2009, the BREAST-Q Reduction Module, a comprehensive, reliable, and statistically validated patient-reported outcome instrument, was introduced. For our study, we utilized items that assessed psychosocial, sexual, and physical well-being, as well as satisfaction with the breasts. These items were specifically designed to detect clinically meaningful changes [10]. To assess patient satisfaction and quality of life one year after surgery, we utilized electronic pre- and postoperative BREAST-Q modules to gather data from enrolled patients. The pre- and postoperative Breast-Q scores were compared using a paired t-test. A *p*-value of less than 0.05 was considered statistically significant. Additionally, we conducted an analysis of NAC sensitivity before surgery and at the two-year follow-up by employing transcutaneous mechanical stimulation of the skin. The sensitivity of the NAC was evaluated by two neurophysiopathologists at our institute, who recommend analyzing nipple and areola sensitivity using this method, both before and two years after the surgical procedure. They systematically transmitted these study data to our medical secretary, who was responsible for creating the study database. To assess interrater reliability, Cohen’s kappa coefficient (for two raters) was used. Mechanical threshold assessment was conducted using Semmes-Weinstein monofilaments. This test evaluates the functioning of mechanosensitive Aβ fibers at a low threshold. During the test, participants were seated with their breasts exposed and were instructed to close their eyes to prevent visual cues while the fibers touched the center of the nipple. They were then asked to verbally report when they felt mechanical stimulation. The mean mechanical thresholds before surgery and at the two-year postoperative mark were compared using the t-test. A p-value of < 0.05 was considered statistically significant. The clinical population’s data and characteristics were analyzed by first checking for normal distribution using the Shapiro–Wilk test, followed by using the Student t-test and Fisher’s exact test. Demographic characteristics were presented as mean and standard deviation (SD) for continuous variables, and as percentage for categorical variables. The associations between clinical variables (BMI, sternal notch to nipple distance, comorbidities, age, and smoking) and any adverse outcomes were evaluated using chi-square and Fisher’s exact tests, with results considered significant at *p* = 0.05. For each patient, data were collected from both paper and electronic medical records. This included their date of surgery, date of birth, weight, height, presence of hypertension (HTA), history of past or current smoking at the time of the first consultation, presence of diabetes, length of hospital stay, resection weight on each side, postoperative drainage through drains, postoperative complications, and duration of wound care consultations.

Specifically, to evaluate the occurrence of postoperative hematoma and other complications with or without the use of suction drains, patients were randomly assigned to either the suction drains group (SDG) or the no suction drains group (NSDG). Randomization was performed using STATA to divide the patients into two equal groups and was employed to minimize selection bias, to ensure that each patient had an equal chance of being assigned to either group and to achieve two equally sized groups. Patients were assigned to groups based on a random assignment using the randuniform function in STATA.

We also assessed the number of patients who underwent pregnancy after the procedure and determined whether they were able to breastfeed.

The operative time was directly collected from the anesthesia records. The characteristics of the patients are presented in Table 1. The duration of drainage, length of hospital stay, operative time, complication rate, and follow-up duration in wound care consultations (outpatient controls until complete wound healing) were analyzed using the ANOVA test, categorized based on the amount of tissue resection ( < 500 g, between 500 and 1000 g, and >1000 g). The authors took full responsibility for data integrity and confidentiality. The statistical analysis was conducted using STATA version 17 (StataCorp LLC, USA).

**Table 1:** Patients characteristics

Characteristics (*n* = 912)	Mean ± SD	Median (IQR)	Min	Max
Age	45.44 ± 13.08	34.78 (27,13 ; 61.97)	18.96	70.95
Height	1.64 ± 0.06	1.64 (1.59 ; 1.68)	1.49	1.78
Weight	71.28 ± 9.46	70 (63 ; 76)	50	97
BMI	26.58 ± 2.73	26.22 (24.58 ; 28.30)	19.72	32.21
Jugular-nipple distance, cm*				
RB (right breast)	RB 35.58 ± 14.73	35.12 (20.3 ; 50.31)	20.2	51.1
LB (left breast)	LB 34.52 ± 11.58	34.23 (20.1; 48.78)	20.01	49.17
Nipple elevation, cm				
RB (right breast)	RB 24.21 ± 6.2	25.4 (5.2 ; 30.21)	5.21	31
LB (left breast)	LB 24.13 ± 7.1	24.8 (5.1 ; 29.11)	5.1	29.2
Comorbidities	No. Patients		Percentage	
Old smoking	123		13.48	
Active smoking^x^	97		10.63	
Diabetes	21		2.30	
Hypertension	71		7.78	

^*^Distance between the jugular fossa and the nipple

^*x*^ current smoking at the time of the first consultation

## Results

**Fig. 2:**
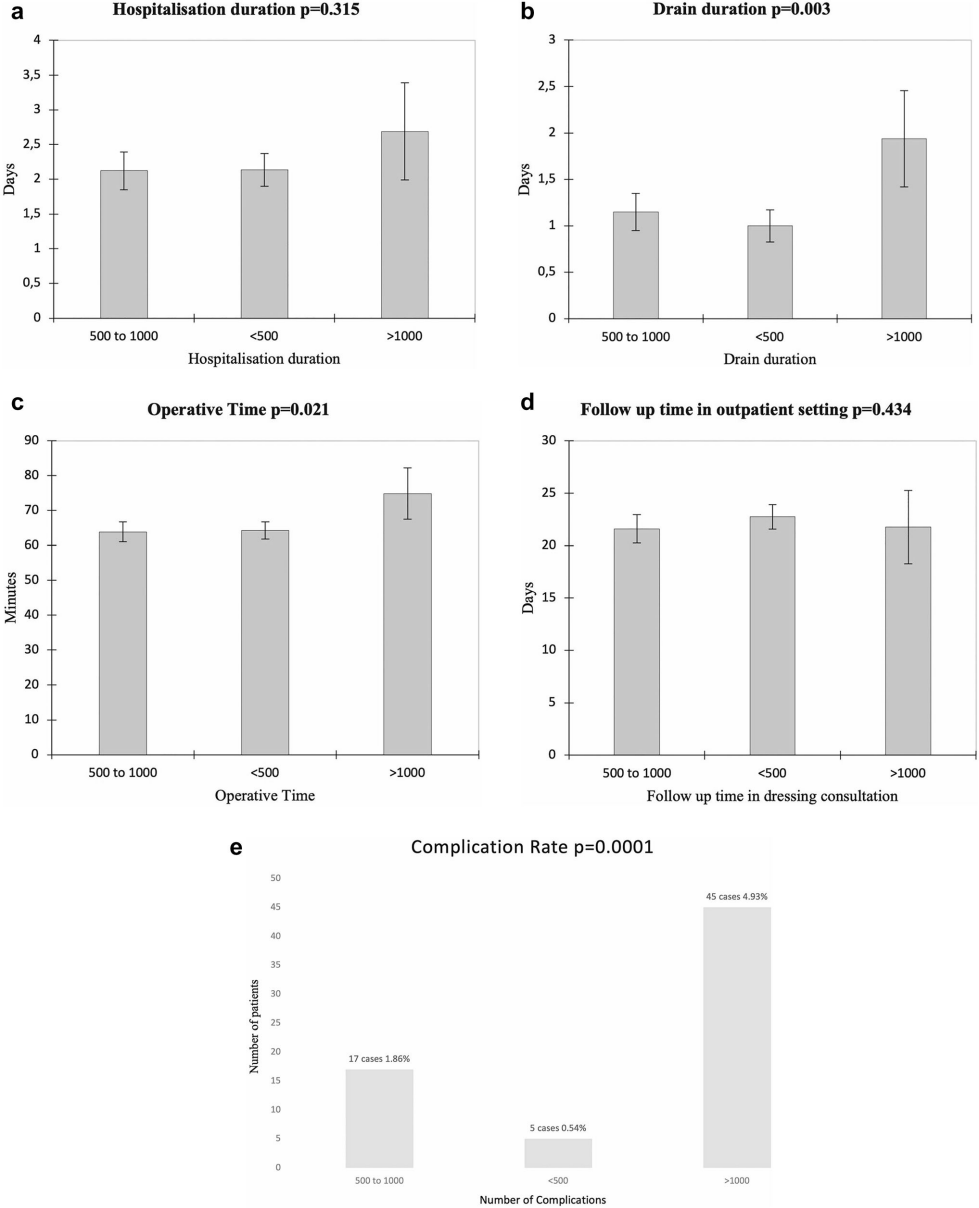
ANOVA test results (**a**-**e**). Drainage duration (for SDG patients), length of hospital stay, operative time, complication rate, and outpatient follow-up time (for all patients) based on the amount of tissue resection ( < 500g, between 500 and 1000 g, and > 1000 g)

We conducted a prospective study on 1003 patients who sought breast reduction surgery for large and ptotic breasts at our university hospital between November 2012 and July 2020. Ninety-one patients dropped out of the study due to non-compliance with follow-up visits for outcome assessment and were subsequently excluded from the analysis. As a result, assessments were conducted on a total of 912 patients (Table 1). The durations of hospitalization, drainage durations, resection weights, operative time, and hematoma rates are summarized in Table 2. Statistically significant differences were observed between patients who had suction drains and those who did not, regarding hospitalization days (*p *= 0.0002), as SDG patients were discharged after drain removal. However, no significant differences were observed in terms of the postoperative hematoma rate and occurrence of other complications between the two groups (*p* > 0.05). The average operative time was 62.12 ± 10.3 minutes, with a minimum of 41 minutes and a maximum of 115 minutes, including the application of an elastocompressive dressing. Patients were monitored through outpatient nurse consultations for an average duration of 21.7 ± 2.4 days after the surgery. Beyond this period, wound healing was complete for patients who did not develop postoperative complications. The mean follow-up period of patients enrolled in this study was 31.21 ± 9.11 months. The ANOVA test results revealed no statistically significant differences in the duration of hospitalization or follow-up duration in outpatient nurse consultations (outpatient controls until complete wound healing) among the subgroups based on the amount of tissue resection ( < 500 g, between 500 and 1000 g, and > 1000 g). However, significant differences were observed for the duration of drainage (*p* = 0.003), operative time (*p* = 0.021), and complication rate (*p* = 0.0001) (Figure 2). The overall rate of complications was 7.34%. The complications were categorized as major and minor. Major complications included those requiring a second surgical intervention, such as hematoma or deep abscess requiring drainage, necrosis of the areola-nipple complex, and the need for blood transfusion. Minor complications included seromas, minor infections requiring antibiotic treatment, wound dehiscence requiring prolonged dressings, and minor wound dehiscence with mild discharge. The distribution of complications is presented in Table 3. Only 61 minor complications were recorded. Ten cases of minor infections treated with antibiotics resulted in wound dehiscence exceeding 3 cm, and one case resulted in minor wound dehiscence with mild discharge. Each patient was only counted once. In our series, no cases of areola-nipple complex necrosis occurred.

**Table 2:** Procedure-related data

Patients	Suction drains group n = 456				No suction drains group n = 456				
	Mean ± SD	Median (IQR)	Min	Max	Mean ± SD	Median (IQR)	Min	Max	*p*
Hospitalization days	2.81 ± 0.90	3 (2 ;3)	1	3	1.51 ± 0.5	2 (1 ;2)	1	2	.0002
Drains (days) *	1.5 ± 0.71	1.5 (1 ;2)	1	3	na	na	na	na	na
Resection weight (right)	542.58 ± 271.80	458 (335 ;679.5)	353	2021	545.81 ± 11.90	461(335;679.5)	364	1899	
Resection weight (left)	546.70 ± 277	465 (340 ; 688)	355	2037	546.70 ± 277	470 (352; 671)	369	2041	0.5
Operative time (min)	64.75 ± 15.11	62 (54 ;73)	41	115	61.12 ± 11.23	61 (51 ;69)	42	113	0.3
Postoperative hematoma	na	na	1	6	na	na	1	5	0.6
Follow-up time (months)	31.22 ± 7.09	24 (19 ; 27)	24	47	31.21 ± 9.11	26 (118 ; 29)	28	49	0.1

^*^ Drainage duration

**Table 3:** Complications

	No. Patients 912	Percentage
Total complications	67 36 SDG, 31 NSDG^x^ p > 0.05	7.34
Major complications	6 4 SDG, 2 NSDG^x^ *p* > 0.05	0.65
Hematoma requiring surgical drainage	5 3 SDG, 2 NSDG^x^ *p* > 0.05	0.54
Major infections	0	0.00
Severe blood loss requiring transfusion: Hb < 7gr/dL	1 SDG, *p* = NA	0.10
NAC necrosis	0	0.00
Minor complications	61 32 SDG, 29 NSDG^x^ *p* > 0.05	6.68
Seroma	11 6 SDG, 5 NSDG^x^ *p* > 0.05	1.20
Minor infections	13 7 SDG, 6 NSDG^x^ *p* > 0.05	1.42
Wound dehiscence > 3cm	10 5 SDG, 5 NSDG^x^ *p* > 0.05	1.09
Wound dehiscence < 3cm with discharge	27 14 SDG, 13 NSDG^x^ *p* > 0.05	2.96

^x^NSDG No Suction drains group

SDG Suction drains group

NA = not applicable

At the 2-year follow-up, nipple–areola sensitivity was fully restored in all 912 patients. There were no statistically significant differences observed between the average preoperative and postoperative mechanical thresholds at the 2-year mark. The mean mechanical threshold before surgery was recorded as 13 ± 1.03 mN, while the mean mechanical threshold two years after surgery was 13 ± 0.8 mN (*p* = 0.3). The Kappa result was 1.00, indicating perfect agreement between the two neurophysiopathologists who assessed NAC sensitivity.

**Fig. 3:**
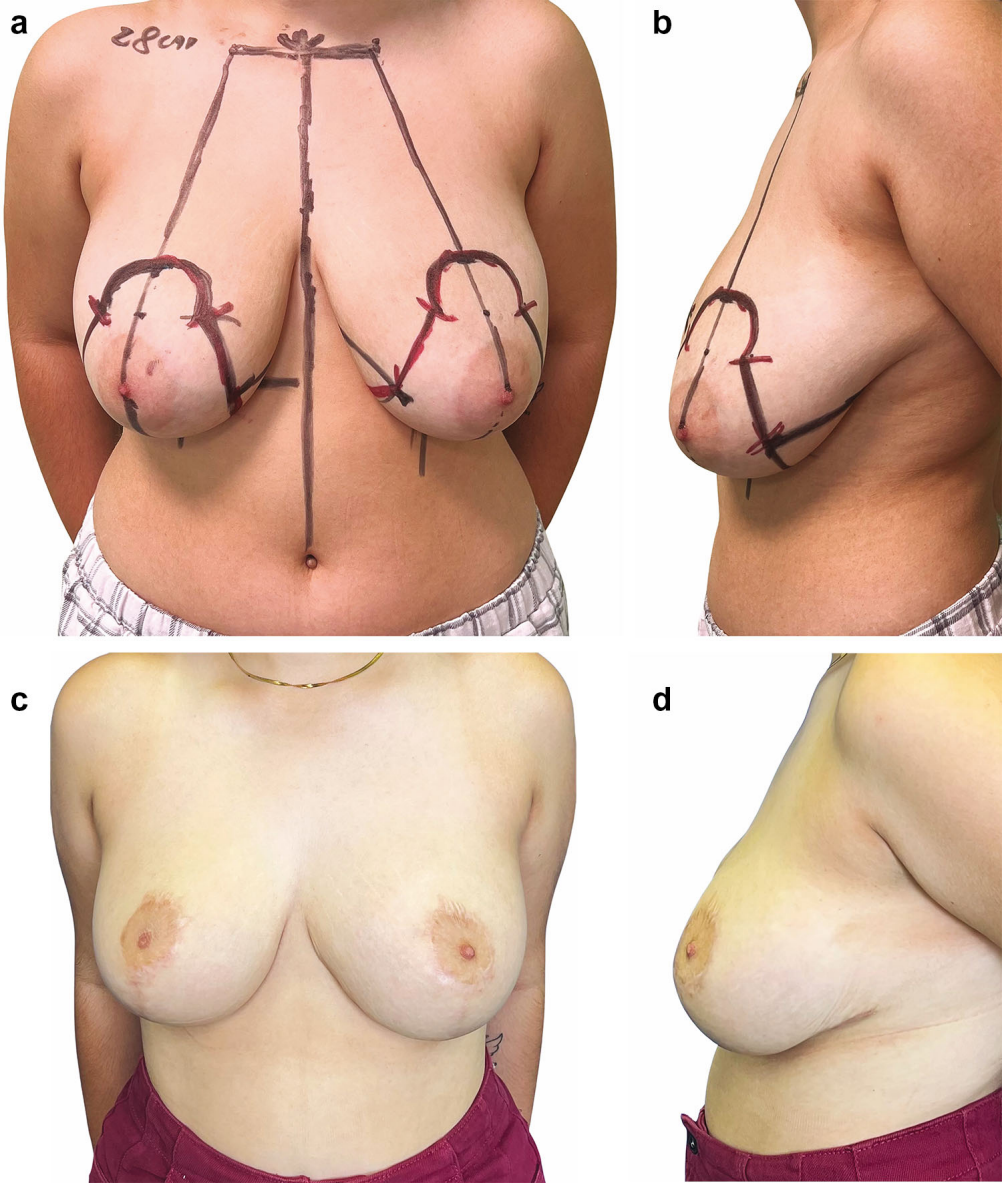
Preoperative (**a**-**b**) and one-year postoperative (**c**-**d**) appearance of a 29-year-old young woman

**Fig. 4:**
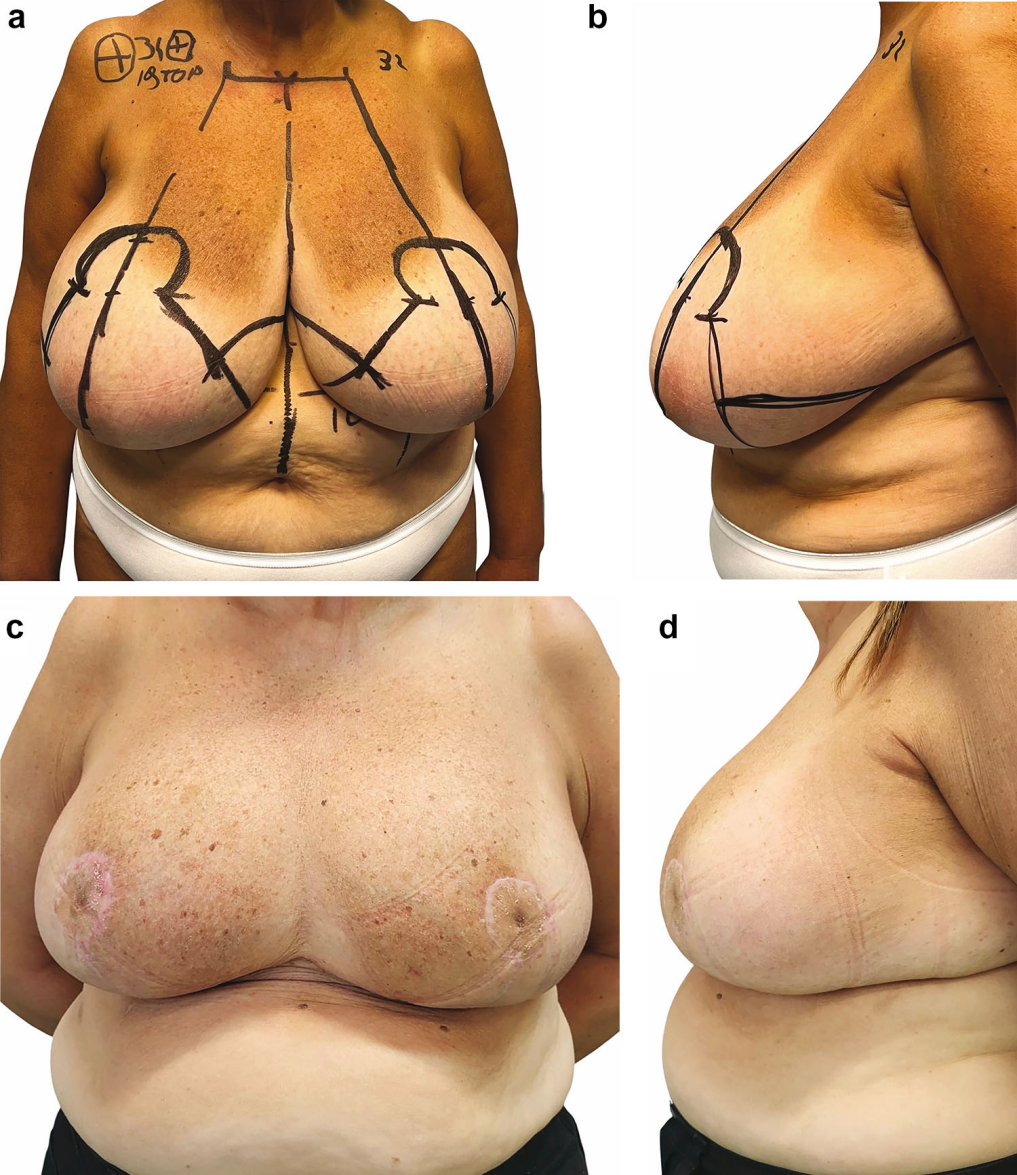
Preoperative (**a**-**b**) and one-year postoperative (**c**-**d**) appearance of a 62-year-old woman. This patient, who had previously experienced severe back pain and had never ventured to the beach before the surgery, expressed profound satisfaction with the results. Furthermore, she had received comprehensive information regarding the potential for less aesthetically pleasing scars. Consequently, in this instance, the advantages gained significantly surpassed the patient’s initial expectations. Remarkably, she barely noticed the periareolar scar outcomes, which were admittedly not very aesthetically pleasing. The postoperative symmastia effect (**c**) can be attributed primarily to the anatomical configuration of the patient’s breast, and its presence can be anticipated in the preoperative images, particularly when examining the upper medial area of both breasts (**a**). After the procedure, the patient expressed extreme satisfaction with the outcome and significant improvement in chronic back pain caused by the weight of her breasts

**Fig. 5:**
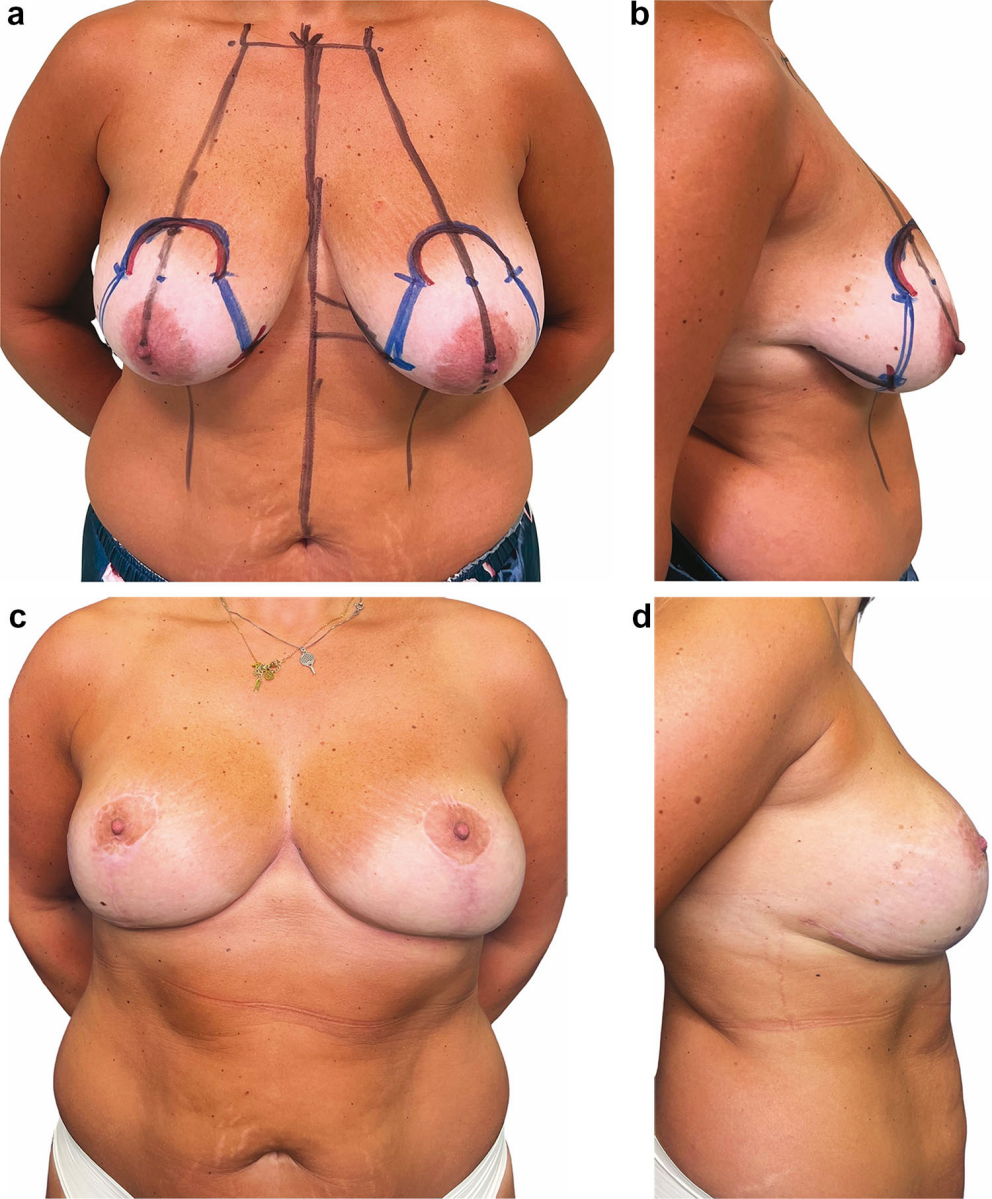
Preoperative (**a**-**b**) and one-year postoperative (**c**-**d**) appearance of a highly active 45-year-old woman. After the procedure, the patient expressed extreme satisfaction with the outcome

A very interesting finding was that out of the 912 treated patients, 47 became pregnant and gave birth during the follow-up period (5.15%). Among these patients, all experienced lactation with secretion from both breasts, and 43 patients breastfed their infants. The remaining 4 patients chose not to breastfeed voluntarily. The evaluation of patient satisfaction using the Breast-Q questionnaire is presented in Table 4 and demonstrates high satisfaction across all assessed domains. All patients reported being very satisfied after surgery, with a statistically significant difference observed between pre- and postoperative scores (*p* < 0.001). In our series, no correlation was found between resection weight and the Breast-Q outcome when performing a Pearson test (*p* > 0.05). The aesthetic outcome in terms of shape and symmetry was satisfactory and stable over time (Figure 3, 4, 5). We achieved a natural appearance of the breast, resulting in high patient satisfaction (mean postoperative Satisfaction with Breast score: 79.26 ± 10.99).

**Table 4:** Baseline and one-year postoperative BREAST-Q Reduction Module Scores

Scale (*n* = 912)	*Baseline* Mean ± SD	*Baseline* Median(IQR)	*Post-op* Mean ± SD	*Post-op* Median(IQR)	*P* value
Psychosocial well-being	50.6 ± 15.3	58(48 ;65)	85.20 ± 9.75	88 (78 ;93)	0.0002
Sexual well-being	45.21 ± 12.32	48 (40 ;52)	75.71 ± 13.67	76 (65 ;82)	0.0001
Physical well-being	48.4 ± 12.1	50 (38 ;58)	83.26 ± 12.82	82 (72 ;90)	0.0003
Satisfaction with breasts	35.7 ± 11.41	35 (30 ;40)	79.26 ± 10.99	75 (70 ;86)	0.0003

Out of the total, 97 patients were active smokers during their initial consultation. However, they tested negative for urine cotinine both before and after the surgery, showcasing a remarkable level of adherence to our guidelines.

Univariate analysis revealed an association between current smoking at the time of the first consultation and an increased risk of wound dehiscence > 3 cm (*p* = 0.018). However, no significant associations were found between the other variables studied and adverse outcomes

## Discussion

Reduction mammaplasty is a commonly performed procedure, with numerous techniques today available [4–7, 12, 13]. As of now, the series of patients presented in our study, consisting of 912 patients, appears to be the largest ever reported in the literature of a single center [1–35].

The rate of complications reported in the literature varies significantly. However, the most frequently encountered complication remains wound dehiscence. Our dehiscence rate of 4.05% (2.96% minor dehiscence and 1.09% dehiscence greater than 3cm) may appear significant, but it aligns with the findings reported in the literature. Klinger et al. [6] reported a wound dehiscence rate of 5.1% in their series of 823 patients. Similarly, Simpson et al. [8] found a scar complication rate of 4.8%. They identified smoking and the use of preoperative corticosteroids as the main contributing factors. Most of these minor dehiscences were observed at the junction where the vertical scar intersects with the horizontal scar, known as the T-junction. Certain studies have reported a dehiscence rate exceeding 8% [15, 16]. In terms of hematomas, our rate of 1.2% also aligns with the findings reported in the literature. Delong et al. [17] demonstrated a similar rate of around 1% in their series. The methodology outlined in our study marks an advancement from Hall-Findlay’s technique [33], characterized chiefly by the broader design of our pedicle. Notably, we emphasize the preservation of a more extensive dermal tissue area (illustrated as point A to point C in Figure 1b). Importantly, we consistently refrain from detaching the pedicle from the pectoralis major muscle’s fascia. In the Hall-Findlay approach, the inner segment of the pedicle concludes at the midpoint of the vertical axis, whereas our technique extends it to the lower end (point C in Figure 1b). Hamdi’s study encompassed 110 patients who underwent his Septum-Based Mammaplasty [19]. However, his pedicle dimensions differ significantly from those elucidated in our study, notably being narrower. Similarly, Van Deventer [34] introduced a technique resembling ours, featuring a patient cohort of 106 individuals who underwent the posteroinferomedial pedicle technique, distinct from our superomedial-posterior pedicle. Our technique represents an evolution of Hall-Findlay’s superomedial pedicle approach combined with the central (posterior) pedicle [35]. The central pedicle reduction mammoplasty is recognized for its versatile vascular supply and innervation, enabling the maintenance of nipple sensation with minimal long-term complications and proven preservation of breastfeeding function. Therefore, our technique encapsulates all the advantages of the methods listed above. Notably, the patient series presented in our study includes 912 individuals, indicating what seems to be the most extensive cohort from a single center reported in the literature up to now. Klinger et al reported the rate of partial NAC necrosis of 3% with 0.48% of total necrosis [6] using the superior pedicle technique. With the same method, Uslu et al [20] reached NAC partial necrosis rate of 2.70%, without total necrosis. No NAC necrosis was encountered in our series, whereas this number can reach up to 10.5% in certain series of breast hypertrophy [18]. This is due to the triple vascular supply provided by our technique: two vascular axes related to the superomedial pedicle and a septal vascularization (Figure 1). Thanks to the superomedial-posterior pedicle, even in cases of a significant distance between the jugulum and the nipple (maximum distance in our series of 51.1 cm) and a significant rise of the NAC (maximum nipple elevation in our series of 31 cm), the vascularization and innervation of the nipple–areolar complex are preserved. This also explains the possibility of breastfeeding for patients who have given birth during the follow-up period, as the entire region of the pedicle, including the retro-areolar area, remains unaltered. It is well known that in cases of gigantomastia or extensive resections leading to a significant elevation of the NAC, there may be an increased risk of complications, particularly ischemic events or venous congestion of the NAC. However, by increasing the width and thickness of the pedicle, the risk of these potential complications is reduced (19, 33–35). Nevertheless, in certain situations, the pedicle might become folded and compressed, resulting in decreased circulation. If tension is suspected, surgical techniques aimed at relieving this tension can be employed [34]. Sometimes, a simple removal of sutures can help restore proper circulation to the NAC. With our technique, it is possible to preserve good vascularization and sensitivity of the NAC, regardless of the jugulum-to-nipple distance. This is attributed to the large and broad pedicle, as well as the preservation of the posterior attachment of the pedicle, which enables improved innervation and vascular supply to the nipple–areolar complex [19]. Indeed, the septum, projected opposite the fifth rib, extending from the pectoralis major to the areola, allows for the passage of intercostal perforators and sensory nerves [22].

The utilization of drains is diminishing within our team. This trend is in line with the observations made by several authors who have shown that the presence of drains does not lead to a decrease in the complication rate [23–26]. Specifically, it is firmly established that drains do not serve as a preventive measure against hematomas; rather, they can only provide information about postoperative bleeding. In line with these findings, we noted no notable disparities in postoperative hematoma occurrence and other complications between patients who underwent surgical drain placement (SDG) and those who did not (NSDG).

However, NSDG patients did experience a shorter hospital stay, highlighting an additional benefit. Our study exhibits a significant strength in terms of operative time. The mean duration of the procedure was 62.12 ± 10.3 minutes, which is the shortest among the other studies [1–32]. The majority of studies reported operative times ranging from 2 hours [20, 27, 28] to almost four hours [17–29]. It is widely recognized that an extended operative time is associated with an increased risk of various complications, regardless of whether low or high-intensity electrocautery is utilized [8–31]. We did not observe any statistically significant differences in the duration of hospitalization or follow-up during outpatient nurse consultations among the subgroups based on tissue resection weight ( < 500 g, between 500 and 1000 g, and > 1000 g). However, significant differences were found in drainage duration (*p* = 0.003), complication rate (*p* = 0.0001), and operative time (*p* = 0.021). Specifically, the complication rate and drainage duration exhibited a clear increase when the excised tissue weight exceeded 1000 g, as depicted in Figure 2. In terms of operative time, a larger resection results in a longer procedure duration due to the need for more extensive hemostasis and excision of a greater amount of tissue. Breast reductions have already demonstrated their effectiveness in reducing back pain, headaches, improving sexual function, enhancing quality of life, self-esteem, and aesthetic satisfaction [1]. Our method appears to be faster and safer compared to the others described in the existing literature. The superomedial-posterior pedicle-based reduction mammaplasty is a safe and reliable method. This technique is effective for treating both small hypertrophies and gigantomastia cases. Respecting the septum helps ensure reliable vascularization and innervation of the nipple–areolar complex. Our method represents the fastest approach documented compared to other techniques described in the literature for breast reduction surgery.

## Limitations

An important limitation of our study could be attributed to the lack of a comparative group. Nevertheless, our results showcase the effectiveness of the technique in terms of safety, restoration of areola-nipple complex sensitivity, and patient satisfaction. The results exhibit high statistical significance, allowing us to consider the analyzed outcomes within a series of 912 patients as reliable, even without a control group. Additionally, it is worth noting that the majority of studies in the literature that analyze or describe a surgical technique do not always include control groups. However, the data from such studies are of paramount importance in guiding surgeons toward choosing a method associated with fewer complications. An undoubtedly notable advantage of our study is the substantial number of patients included in the analysis.

### Supplementary Information

Below is the link to the electronic supplementary material.

Supplementary file1 (MOV 497299 KB)

## Declarations

### Human and Animal Resource

All procedures in the study involving human participants have been performed in accordance with the ethical standards of institutional and/or national research committees and with the 1964 Helsinki Declaration and its later amendments or comparable ethical standards.
